# Spasticity and Abnormal Tone Regulation After Spinal Cord Injury: Mechanisms and the Effects of Neuromodulation

**DOI:** 10.3390/biomedicines14061348

**Published:** 2026-06-15

**Authors:** Joshua Ceisler, Nilanjana Datta, Pedro P. Saraiva, James D. Guest

**Affiliations:** 1 The Neuroscience Graduate Program, University of Miami Miller School of Medicine, Miami, FL 33136, USA; jac89459@miami.edu; 2 The Miami Project to Cure Paralysis, and the Lynn Rehabilitation Center, University of Miami Miller School of Medicine, Miami, FL 33136, USA; nxd693@med.miami.edu (N.D.); pmpsaraiva@med.miami.edu (P.P.S.); 3 Department of Neurological Surgery, University of Miami Miller School of Medicine, Miami, FL 33136, USA

**Keywords:** spinal cord injury, spasticity, muscle tone, neuromodulation, transcutaneous spinal cord stimulation, corticospinal tract, reticulospinal tract, neuroplasticity

## Abstract

Spinal cord injury (SCI) is frequently accompanied by abnormal muscle tone and spasticity, which impair voluntary motor control, mobility, and quality of life. Although classically defined as velocity-dependent hyperreflexia, tone abnormalities after SCI encompass a broader spectrum, including sustained muscle activation, co-contraction, clonus, and non–velocity-dependent resistance to movement. These manifestations arise from distributed changes across spinal and supraspinal motor systems. At the segmental level, SCI induces maladaptive plasticity involving motoneurons, interneurons, sensory afferents, and muscle, including dysregulated persistent inward currents, altered inhibitory neurotransmission, afferent hyperexcitability, synaptic reorganization, and structural muscle remodeling. In parallel, supraspinal adaptations—including cortical motor map reorganization, reduced intracortical inhibition, corticospinal–reticulospinal imbalance, loss of monoaminergic modulation, and altered brainstem and cerebellar regulation—further amplify spinal circuit gain and impair inhibitory control of tone. Current pharmacologic treatments largely suppress symptoms without addressing these underlying circuit changes, while invasive neuromodulatory strategies are limited by surgical risk or state-dependent effects. This review synthesizes emerging insights into the multilevel mechanisms regulating abnormal tone after SCI and examines neuromodulatory approaches targeting spinal and supraspinal networks. Particular attention is given to transcutaneous spinal cord stimulation (TcSCS), a non-invasive method capable of modulating segmental reflex circuits and descending control pathways. Advances in transcriptomic and epigenetic profiling may further enable mechanism-based therapies and biomarker-guided strategies for treating spasticity.

## 1. Introduction

Spinal cord injury (SCI) commonly impairs voluntary motor control and coordinated muscle activation. The loss of motor control results from both paralysis and excessive muscle activity, as evidenced by spasticity, clonus, and joint contractures. In this article, we focus on abnormalities of tone following SCI, their mechanisms and neurophysiology, and the neural substrates through which targeted interventions can better regulate excess tone, with a focus on emerging neuromodulatory approaches. As discussed, multiple sources of abnormal tone have been underappreciated due to a focus on strict definitions of “spasticity”. Spasticity is most strongly associated with spinal cord lesions that partially interrupt descending inhibitory pathways while preserving segmental reflex and motor circuitry below the lesion. This pattern is especially typical of incomplete cervical or thoracic SCI but is also common in multiple sclerosis with spinal cord involvement and in degenerative cervical myelopathy.

Muscle tone, defined as the resistance opposing passive stretch in a relaxed muscle, is essential for maintaining posture, facilitating movement transitions, and providing foundational neuromuscular control [1]. Physiological tone results from interactions between intrinsic muscle properties, spinal reflexes, and descending supraspinal regulation. Spasticity, defined as velocity-dependent increases in muscle tone with exaggerated tendon reflexes due to hyperexcitability of the stretch reflex, can significantly impair function and quality of life after SCI [2,3,4,5]. The prevalence of spasticity across reports is approximately 70% [6], with “problematic” spasticity in 35% [7]. Triggers for hyperreflexia can be minimal yet can result in whole-body spasms; heightened tone can limit joint range of motion (ROM), thereby restricting voluntary movements. Individuals with upper motor neuron SCI, as compared to those with lower motor neuron SCI (whose muscles may be flaccid), frequently exhibit excessively sustained contractions, co-contraction of agonist and antagonist muscles, and fluctuating tone states. In SCI clinics, one often also sees individuals with very elevated tone that resembles other neurological conditions like stroke, Parkinson’s disease, and multiple sclerosis. This tone may appear as resistance to motion that is not clearly velocity-dependent. Patient-reported symptoms, including stiffness, involuntary movements, and fluctuations in tone, are difficult to capture with standard assessments. Thus, the clinical distinction between spasticity and hypertonia is often unclear in practice.

While velocity dependence arises from the role of the muscle spindle as a feedback regulator of muscle stretch and its primarily monosynaptic circuits, other tone abnormalities arise from the brain and brainstem, as is observed after stroke. Thus, improved classification of tone abnormalities is needed, ranging from heightened resting tone to inefficient and restricted movements to actual spasms and unwanted joint oscillations, such as clonus.

Classically defined spasticity is observed across SCI severities, particularly in incomplete injuries [8,9]. Anti-spasticity treatments, including stretching and medications, tend to have limited efficacy and persistence. The significance of tone problems and their resistance to treatment underscores the need for a better understanding of the mechanisms of abnormal tone.

## 2. A Conceptual Framework to Understand Abnormal Tone After Spinal Cord Injury

**Figure 1:**
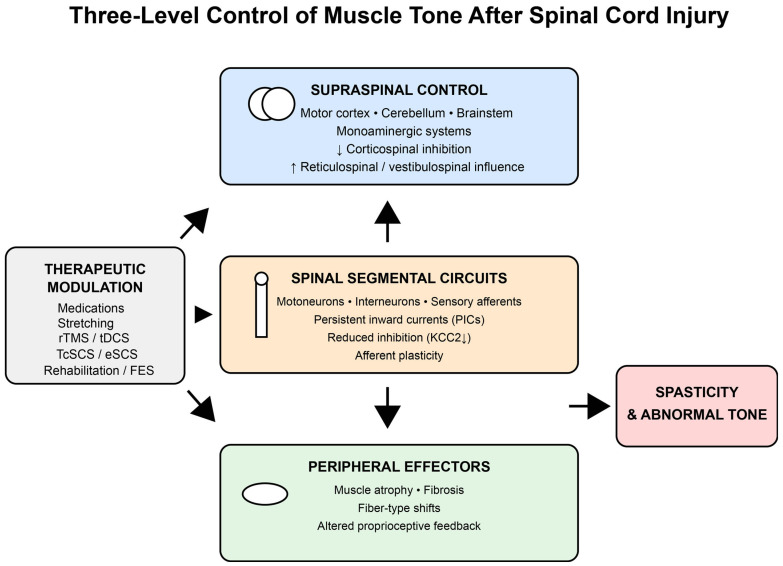
Spasticity and abnormal tone arise from interactions among supraspinal control, alterations in spinal segmental circuits, and changes in peripheral effectors as indicated by arrows. Different therapies may address each component. Figure 1 was designed with assistance using Claude 3.7 Sonnet and Microsoft Powerpoint (16.103, 2025) with final editing in Adobe Illustrator (30.21,2026).

Descriptions of physiology often require simplifying concepts to dissect the complex milieu of simultaneous actions. One helpful approach is to separate tone-regulating mechanisms into segmental and other mechanisms, such as supraspinal and intersegmental factors (Figure 1).

We define a spinal cord segment as: the discrete set of sensory and motor neurons that give origin to the respective roots, and the gray matter interneurons and supporting cells that arise in that segment. Some neurons within the segment may participate in intersegmental integration as part of the propriospinal system, whose spinal cord neurons and intersegmental axons connect between segments [10].

A segmental approach to understanding abnormal tone arises from neurodevelopment during which the spinal cord becomes segmentally organized from paraxial somites and sclerotomes that dorsally and ventrally give origin to the segmental motor neurons and the neural crest that provides the afferent dorsal root ganglion sensory neurons at each level [11,12] as the basis for a rostral to caudal system of segmental reflexes.

Segmental dorsal and ventral interneurons are essential for left-right coordination, movement pattern generation, and excitatory and inhibitory balance. They integrate incoming sensory information with motor neuron outputs. The propriospinal interneurons that arise in the fetal period integrate across multiple spinal levels to serve coordinated intentional movement and reflex responses [13,14]. Axons of the major tracts arrive at the developed segments in sequence, with reticulospinal (RST) first and corticospinal (CST) last, and form synapses on interneurons and motor neurons that mediate motor control.

The term “supraspinal” refers to the conscious and automatic control systems of the brain, brainstem, and cerebellum that regulate spinal cord output. These systems govern essential life-supporting functions such as respiration and blood pressure control, coordinate rapid responses to environmental threats, and enable intentional, complex voluntary behaviors (e.g., playing the piano). Supraspinal control encompasses the motor and sensory cortices, subcortical nuclei, including the basal ganglia, the cerebellum, and brainstem centers, which communicate with spinal segments via descending and ascending white matter pathways that surround the intrinsic spinal cord circuitry.

### Altered Segmental Function After SCI

SCI is usually a focal injury in which a portion of the segmental neurons and the ascending and descending tract axons are lost. This results in much of the neural circuitry below the focal injury level being relatively intact but lacking the prior regulating supraspinal connections. Because of connection loss, the preserved denervated circuits undergo extensive reorganization and adaptations that underlie abnormal tone and spasticity.

## 3. Loss of Neurons

SCI acutely damages motor neurons, interneurons, glia, and tissue components such as blood vessels in the region of the injury. The pattern of cell loss is often observed to have a biconical graded distribution with maximum injury at the injury epicenter that tapers off in the adjacent rostral and caudal segments [15]. Mechanical axonal injury, hemorrhage, and ensuing intracellular and extracellular edema, along with severe membrane dysfunction, cause neuronal and glial death within minutes to hours via necrosis, apoptosis, and other cell death mechanisms [16]. In rodent models, apoptotic motor neurons death signaling peaks at 8 h post-injury [17], with graded cell loss extending beyond the lesion epicenter [18]. Lost motor neurons are not replaced.

## 4. Neuroplasticity

Neuroplasticity related to spasticity encompasses injury- evolution and activity-dependent changes in neural circuit structure and function following SCI that alter synaptic connectivity, intrinsic neuronal excitability, and network dynamics. After SCI, these processes include axonal sprouting of sensory afferents, synaptic remodeling within spinal interneuron networks, alterations in motoneuron intrinsic properties such as persistent inward currents (PICs), shifts in inhibitory neurotransmission, and reorganization of descending motor pathways. While some forms of neuroplasticity support recovery of voluntary function, others promote an imbalance between excitation and inhibition within spinal and supraspinal motor networks, reinforcing hyperexcitable circuit states that underlie spasticity and abnormal muscle tone.

## 5. Deafferentation and Synaptic Reorganization

When segmental neurons lose synaptic inputs after SCI, they become deafferented, and the ensuing neuroplastic reorganization is a major contributor to spasticity and increased muscle tone [19,20]. Surviving neurons undergo structural and functional remodeling in response to the altered extracellular milieu, including changes driven by neurotrophic factors such as brain-derived neurotrophic factor (BDNF) [21]. Newly available synaptic sites are competitively reoccupied by sprouting terminals [22], reshaping neuronal responsiveness through both permissive and restrictive cues. Astrocytic and neuronal secreted molecules act as synaptogenic attractants, promoting synapse formation and maturation; key astrocyte-derived factors include thrombospondins, hevin, and glypicans [23]. In contrast, synaptic constraints comprise molecular and environmental signals that limit where and when new connections stabilize, including repulsive guidance pathways such as ephrin–Eph signaling, chondroitin sulfate proteoglycan–rich extracellular matrix, and the evolving inflammatory milieu [24].

### 5.1. Motoneuron and Interneuron Plasticity

The motor neuron, its axon, and the associated motor unit are the final common pathway of neuromuscular control. Motor neurons normally utilize persistent inward currents (PICs) because the spinal system requires intrinsic amplification to turn brief, sparse, or noisy synaptic input into sustained, stable, and graded motor output. PICs become dysregulated after SCI, contributing to spasticity [25]. In the chronic SCI state, motor neurons exhibit dysregulated PICs and plateau potentials, thereby elevating the membrane potential and prolonging depolarization in response to input [26]. This is mediated by sodium and calcium influx [27] and correlates with observed hyperexcitability and spastic behaviors in people with SCI [28].

Interneurons are critical integrators of excitation-inhibition balance. During neural development, the activity level influences whether dorsal interneurons adopt a glutamatergic(excitatory) or GABAergic(inhibitory) phenotype [29]. After SCI, neurotransmitter phenotype switching in spinal interneurons is driven primarily by activity-dependent homeostatic plasticity triggered by the abrupt loss of descending monoaminergic and excitatory inputs [30]. This reduction in synaptic drive reduces intracellular calcium signaling, thereby activating gene programs that shift transmitter expression, with vGlut2+ excitatory neurons acquiring GABA expression [31] and increased GAD67 enzyme expression observed in the injured feline spinal cord [32], suggesting compensatory inhibitory upregulation [33]. Concurrent circuit reorganization, including propriospinal sprouting and changes in afferent input patterns, further reinforces transmitter identity adjustments. While these changes may partially limit hyperexcitability, they can also inhibit movement initiation.

Other molecular changes may instead potentiate hyperexcitability. Following SCI, the abrupt loss of descending inhibitory input is accompanied by increased levels of neurotrophic and inflammatory mediators in the dorsal horn, including BDNF and cytokines such as TNF-α and IL-1β [34,35]. These signals can disrupt chloride homeostasis by reducing expression of the potassium–chloride cotransporter KCC2, leading to elevated intracellular chloride concentrations. As a result, activation of GABA_A and glycine receptors—normally inhibitory—becomes less effective at hyperpolarizing neurons and may instead produce depolarizing responses, thereby increasing network excitability. The relationship between BDNF and KCC2 after SCI appears to be complex and time-dependent. Experimental sequestration of endogenous BDNF signaling immediately after injury (e.g., using TrkB-Fc) prevents the early downregulation of KCC2, indicating that endogenous BDNF contributes to this acute decrease in chloride extrusion capacity. In contrast, in the post-injury state, experimentally delivered (exogenous) BDNF can increase KCC2 expression and restore inhibitory function [36]. Thus, BDNF appears to regulate KCC2 in a context-dependent manner after SCI rather than exerting a simple unidirectional effect. The changes in ionic balance may amplify excitability and promote spasticity [36,37].

### 5.2. Changes in Dorsal Root Ganglion Sensory Neurons

Sensory afferents are normally the primary source of spinal reflex activity. After SCI, primary afferent neurons undergo molecular and structural changes that drive hyperexcitability of spinal circuits and contribute to spasticity and neuropathic pain. Dorsal root ganglion (DRG) neurons below the lesion undergo central and peripheral sprouting [38] and altered expression of ion channel and axonogenesis genes [39].

The afferent sensory neurons become intrinsically hyperexcitable due to upregulation of voltage-gated Na^+^ channels (NaV1.3, NaV1.7) and downregulation of K^+^ channels, leading to spontaneous and ectopic firing that tonically excites dorsal horn neurons and motoneurons [40,41,42]. Concurrently, Aβ low-threshold mechanoreceptors sprout into nociceptive laminae, and C-fibers increase expression of substance P, CGRP, and BDNF, producing exaggerated excitatory drive and sensory–motor coupling [37,38,43]. Proprioceptive afferents (Ia, Ib) also form new collaterals in motor laminae, enhancing monosynaptic reflex gain and contributing to spasms [44,45]. In rodent experiments, small DRG neurons showed sustained increases in spontaneous firing as early as 3 days post-SCI and persisted for up to 8 months [41].

Together, these maladaptive afferent and microcircuit changes transform normal sensory inputs into sustained excitatory drive, forming the substrate for persistent reflex activity and hyperreflexia after SCI. This sensory reorganization alters integration at spinal and supraspinal levels, abnormally modifying reflex gain and excitability.

### 5.3. Emergence of Silent Synapses After SCI

After SCI, the “unmasking” of silent synapses—glutamatergic connections that express NMDA but not AMPA receptors—contributes to spinal hyperexcitability and spasticity by converting previously inactive inputs into functional excitatory synapses. In the intact spinal cord, these synapses remain quiescent because Mg^2+^ blocks the NMDA receptor channel at resting potentials. Following injury, elevated extracellular glutamate, loss of inhibitory tone, and prolonged depolarization relieve this block, while activity-dependent and neuroinflammatory signaling promote the insertion of AMPA receptors into postsynaptic membranes. BDNF and TNF-α released from activated microglia and astrocytes trigger AMPAR trafficking via the PI3K–Akt and CaMKII pathways [37,43], transforming formerly silent synapses into active excitatory synapses. Electrophysiological studies demonstrate enhanced NMDA-dependent excitatory potentials and increased AMPAR-mediated currents in dorsal horn and propriospinal neurons after SCI, [46,47] indicating widespread synaptic unsilencing. While this mechanism can support adaptive reconnection of spared descending or intersegmental propriospinal pathways, it also amplifies excitatory drive to motoneurons and interneurons, fostering persistent reflex activity, clonus, and spasticity.

## 6. Relay Circuits

Following SCI, relay circuits between corticospinal (CST) axons and propriospinal interneurons emerge as a key form of structural and functional reorganization in experimental animals. In incomplete injuries, spared CST fibers sprout onto propriospinal neurons, which have projections below the lesion that can reinnervate motor pools, partially restoring voluntary control [48,49]. However, when descending inhibitory pathways are lost, these spinal intrinsic circuits can become hyperexcitable, contributing to spasticity and exaggerated reflex activity [50]. Increased excitatory convergence from CST, RST, and sensory afferents onto propriospinal neurons, combined with reduced glycinergic and GABAergic inhibition, creates self-sustaining excitatory loops within spinal networks [47,51]. These reorganized propriospinal pathways thus serve a dual role—facilitating adaptive reconnection and recovery when appropriately modulated but promoting maladaptive hyperexcitability and spastic motor output when inhibitory control and synaptic balance are lost. The existence of such circuits is difficult to prove in humans [52], underscoring that much of our theory rests on preclinical studies.

### 6.1. Post-SCI Changes in Muscle and Bone Properties

Skeletal muscles retain the segmental organization of their motor neurons and sensory afferents, forming an integrated neuromuscular unit whose trophic status depends on both neural activity and mechanical loading. After SCI, loss of descending drive, denervation of injury-level segments, and reduction in weight-bearing and stretch stimuli trigger a cascade of catabolic and fibrotic remodeling. Muscle cross-sectional area declines by 45–80% within 6 months of complete SC [53], while intramuscular fat and connective tissue increase threefold within 6 weeks and continue rising for several months [54]. Early transcriptomic changes include extensive extracellular matrix remodeling and fibrosis, with upregulation of proteasome and autophagic pathways, downregulation of PI3K–Akt signaling, and upregulation of fibrosis-associated ECM genes such as *COL1A1* and *TGFβ1* [55].

Denervated muscle fibers undergo a reversal of the fast-to-slow conversion, with a marked shift toward fast glycolytic isoforms, reduced oxidative capacity, and increased fatigability [56]. Over time, muscle stiffness, shortened fascicles, and disorganized sarcomeric alignment exacerbate reflex gain, amplifying tone and spasticity through altered proprioceptive feedback.

Bone undergoes a parallel but distinct degenerative process. Loss of mechanical strain and neuromuscular loading leads to rapid disuse osteopenia, with bone mineral density (BMD) declining by roughly 1% per week during the first months and reaching osteoporotic levels within 2 years [57]. The most pronounced losses occur in the distal femur and proximal tibia, where trabecular bone volume can fall by over 50%. Osteoclastic resorption is driven by elevated RANKL/OPG ratios, systemic inflammation, and vascular dysregulation below the lesion. In parallel, heterotopic ossification (HO)—the ectopic formation of bone within muscle—occurs in up to 20–30% of individuals with SCI [58], producing painful contractures and spastic muscle hardening that further impair rehabilitation.

Together, these musculoskeletal adaptations represent a profound reorganization of peripheral tissue physiology following SCI—an interplay of denervation, disuse, inflammation, and impaired trophic signaling that transforms muscle and bone.

Despite this muscle and bone loss after SCI, several lines of evidence show that exercise and neuromuscular activation can partially reverse these degenerative adaptations. Regular functional electrical stimulation (FES) and resistance training restore contractile loading and reactivate anabolic PI3K–Akt–mTOR pathways, increasing muscle cross-sectional area, reducing intramuscular fat, and improving oxidative enzyme activity [59]. FES-evoked cycling and activity-based locomotor training also promote fiber-type remodeling toward more fatigue-resistant phenotypes and preserve sarcomere organization [60,61]. In bone, mechanical and electrical loading during FES-assisted standing or cycling attenuates bone mineral density loss in the distal femur and tibia by reactivating osteocyte mechanotransduction [62,63]. Exercise also reduces expression of fibrosis-associated genes such as *COL1A1* and *TGFβ1*, thereby limiting extracellular matrix expansion [64,65], and improves systemic metabolic function by enhancing insulin sensitivity and resting energy expenditure [66,67]. FES cycling is correlated with spasticity reduction [68]. Collectively, these studies demonstrate that even in chronically paralyzed muscle and bone, activity-dependent rehabilitation and electrical stimulation preserve tissue structure, promote adaptive remodeling, and mitigate secondary complications.

### 6.2. Altered Supraspinal Organization and Function After SCI

In an analogous manner to the deafferentation changes that occur at and below the injury level, there are many alterations above the SCI level that arise from the loss of ascending sensory system axons and reduced or altered activity in descending axons whose terminals have been severed, or as a result of injury-responsive neuroplasticity.

### 6.3. Cortical Motor Map Reorganization

The cortical representation of the body is dynamic, and following SCI, representations of neuromuscular ensembles are often reduced due to sensory deafferentation, diminished voluntary motor activity, and disuse. However, despite prolonged disconnection from the periphery, cortical motor representations of paralyzed muscles are rarely abolished. Instead, they typically undergo contraction, reduced excitability, and partial remapping, while the core somatotopic organization is preserved even in chronic, motor-complete injuries. Functional neuroimaging and TMS studies demonstrate that attempted or imagined movements of paralyzed limbs reliably activate corresponding regions of the primary motor cortex (M1), indicating preservation of internal motor models and recurrent cortical circuitry [69,70]. Intracortical recordings in individuals with long-standing tetraplegia further show that M1 neurons retain normal directional tuning and muscle-synergy encoding despite decades without descending output [71,72]. Together, these findings indicate that M1 representations remain viable targets for neuromodulation and neuroprosthetic intervention after SCI.

Residual intact connections above the injury may undergo compensatory expansion. Using TMS, Levy et al. demonstrated that muscles rostral to the lesion are represented over enlarged cortical territories compared with controls [73], with similar findings reported in complete thoracic SCI [74]. In incomplete cervical SCI, the cortical representation of the abductor pollicis brevis (APB) is often medially shifted and bilaterally enlarged, potentially reflecting the muscle’s behavioral relevance and frequent use in daily activities [75].

#### 6.3.1. Impact of Rehabilitation on Cortical Representation

Cortical motor maps after SCI remain highly plastic, and targeted, intensive rehabilitation can drive adaptive reorganization in spared circuits. When descending or ascending pathways are preserved, task-specific practice expands the cortical representation of trained muscles, partially reversing injury-related map contraction. Repeated practice—whether voluntary, robotic-assisted, or paired with functional electrical stimulation (FES)—strengthens residual CST and propriospinal pathways, enhances excitatory intracortical connectivity, and re-engages latent circuits in M1, premotor, and supplementary motor areas. Sensory feedback during training is a critical driver of these effects, promoting coherent cortical organization and limiting maladaptive plasticity following deafferentation [76,77].

At the circuit level, rehabilitation strengthens intracortical horizontal connections, reflected by increased short-interval intracortical facilitation and improved coordination between M1 and higher-order motor regions [78]. Task-specific training is thought to reinforce existing corticospinal projections, recruit latent polysynaptic relay pathways (e.g., corticoreticulospinal and propriospinal circuits), and rebalance intracortical excitability through coordinated reductions in inhibitory tone and increases in synaptic efficacy [79]. Collectively, these changes reflect a shift toward network-level optimization rather than simple focal map expansion.

Consistent with these mechanisms, human TMS and fMRI studies demonstrate that structured grasp, reach, or locomotor training in incomplete cervical SCI enlarges the cortical representation of trained muscles, with changes correlating with improvements in voluntary control, grip strength, and dexterity [69,80].

#### 6.3.2. Reduced Intracortical Inhibition and Motor Preparation Deficits

Effective motor control depends not only on the activation of appropriate muscles but also on the suppression of unwanted or poorly timed motor commands. This suppression is mediated in part by intracortical inhibitory circuits, largely composed of GABAergic interneurons that regulate corticospinal output before it reaches the spinal cord [81,82,83]. These inhibitory mechanisms maintain movement precision, prevent antagonist co-contraction, and coordinate voluntary commands with reflex modulation. After SCI, disruption of intracortical inhibition shifts this balance toward excessive or mistimed activation, contributing to abnormal tone and spasticity [84].

Physiologically, short-interval intracortical inhibition (SICI) is reduced in chronic SCI despite preserved corticospinal recruitment curves, indicating selective impairment of inhibitory cortical function [85]. In addition, normal suppression of corticospinal excitability during movement withholding is diminished [86], potentially predisposing individuals to inappropriate or excessive motor output.

### 6.4. Corticospinal–Reticulospinal Imbalance

In the intact nervous system, the regulation of voluntary movement and muscle tone depends on a finely tuned balance between the CST and RST pathways [87,88]. The CST provides precise, fractionated control of distal musculature and also exerts a strong inhibitory influence over spinal reflex circuits, both directly via spinal interneurons and indirectly through modulation of brainstem centers [89]. In humans, the CST also has direct excitatory connections to motoneurons, further contributing to selective motor control. By contrast, the RST, originating in the pontomedullary reticular formation, supports posture, gross limb movements, and rapid, generalized motor responses [88]. Although the RST conveys both excitatory and inhibitory signals, its net excitatory drive to spinal motoneurons is normally constrained by CST and other supraspinal inhibitory influences [90]. Disruption of this balance—through loss of corticospinal inhibition or relative upregulation of reticulospinal output—biases spinal circuits toward hyperexcitability. Consistent with this model, individuals with incomplete SCI and spasticity exhibit reduced voluntary corticospinal drive, lower transcranial motor evoked potential (MEP) amplitudes, and diminished quadriceps strength. A loud sound (startle) can activate the RST. However, the RST may contribute more strongly to motor output after SCT injury [91], an effect that can be measured using the auditory startle response, an evolutionarily conserved brainstem response conveyed via the RST to the spinal cord and terminating in a divergent manner on interneurons. An imbalanced RST drive is implicated as a contributor to spasticity [92].

In addition to changes in reticulospinal drive, alterations in vestibulospinal pathways contribute to abnormal tone and spasticity after SCI. The lateral vestibulospinal tract (LVST) provides strong, tonic excitatory input to axial and proximal limb extensors and is a key component of antigravity and postural control circuits [93]. When descending cortical and midbrain inhibitory influences are reduced by SCI, vestibulospinal output becomes relatively disinhibited, producing an extensor bias, increased stretch-reflex gain, and greater resistance to passive movement [94,95]. This enhanced vestibulospinal drive is reflected clinically by extensor-dominant tone, postural rigidity, and exaggerated reflex responses.

Human neurophysiological studies support this model, demonstrating facilitated vestibulospinal reflexes and abnormal vestibular-evoked myogenic potentials in individuals with SCI, consistent with increased vestibulospinal excitability [96,97]. Vestibulospinal pathways can be probed using trans mastoid galvanic stimulation, which evokes characteristic changes in postural extensor muscle EMG and can reveal residual descending connectivity after injury [98,99]. These responses exhibit prolonged latencies following SCI [95], further indicating altered vestibulospinal processing. Together with reticulospinal disinhibition, these brainstem adaptations create a supraspinal environment that amplifies spinal hyperexcitability and promotes the extensor-dominated tone characteristic of chronic SCI [100].

### 6.5. Loss of Monoaminergic Neuromodulatory Systems and Circuit Gain Modulation After SCI

Descending neuromodulatory systems originating in the brainstem—including serotonergic projections from the raphe nuclei and noradrenergic projections from the locus coeruleus—play a critical role in regulating the gain of spinal motor and sensory circuits [101,102]. These monoaminergic inputs modulate motoneuron excitability in both dendritic and somatic compartments by activating metabotropic G-protein coupled receptors, such as 5-HT_2_ and α_1_-adrenergic receptors, which strongly influence the expression of PICs and thereby shape motoneuron firing behavior. Under normal conditions, this dynamic modulation enhances voluntary motor output while preserving inhibitory control over reflex pathways, preventing excessive or unwanted muscle activity.

Following SCI, disruption of these descending monoaminergic pathways produces profound changes in the regulation of spinal excitability [103,104]. In the acute phase, the loss of serotonergic and noradrenergic tone decreases intrinsic motoneuron excitability, contributing to the transient period of spinal shock and flaccid paralysis. Over subsequent weeks, however, denervated motoneurons and interneurons undergo homeostatic and maladaptive plasticity: PICs re-emerge and become exaggerated due to receptor supersensitivity, altered receptor distribution, and constitutive activity of 5-HT_2_ and α_1_-adrenergic receptors [104,105]. These molecular adaptations markedly increase intrinsic excitability, making motoneurons more likely to depolarize and discharge in response to minimal synaptic input.

This maladaptive gain amplification at the motoneuron level directly contributes to hyperreflexia, clonus, spasms, and velocity-dependent increases in tone characteristic of spasticity. In parallel, the loss of descending monoaminergic modulation disrupts presynaptic inhibition of Ia afferents and diminishes inhibitory control of segmental interneuron networks [103], further promoting excessive reflex responses and sustained muscle contractions. Together, these changes transform a transient post-injury hypoexcitable state into a chronic condition of exaggerated spinal excitability and impaired inhibitory regulation, providing a central mechanistic pathway through which monoaminergic denervation contributes to spasticity after SCI.

## 7. Tone, Spasticity, and the Cerebellum

Through its cerebello–reticulospinal and cerebello–vestibulospinal projections, the cerebellum plays a central role in fine-tuning spinal excitability, scaling reflex responses, and suppressing inappropriate motor output. In the intact system, these pathways provide ongoing inhibitory and modulatory control over brainstem motor centers, ensuring that descending commands remain precisely calibrated to proprioceptive and contextual demands. After SCI, loss of spinocerebellar input deprives the cerebellum of real-time information about limb position, load, and segmental motor output, impairing its ability to predict, filter, and dampen excessive reflex gain. This deafferentation disrupts cerebellar modulation of the brainstem reticular and vestibular nuclei, contributing to maladaptive increases in excitatory reticulospinal and vestibulospinal drive and reduced supraspinal control of γ-motor and spinal interneuronal circuits. In addition, cerebellar changes have been correlated with altered sensorimotor function and chronic pain [106].

At the same time, chronic adaptations within cerebellar and cerebellar–brainstem circuits—including altered activation patterns, changes in Purkinje cell output, and reweighted connectivity—further degrade the cerebellum’s role in regulating segmental excitability. Neuroimaging studies in humans with SCI demonstrate structural atrophy and altered functional connectivity within cerebellar networks [107], and these changes parallel findings in other neurological disorders in which cerebellar dysfunction is associated with hyperreflexia, impaired sensorimotor integration, and elevated tone [108]. A study examining cerebellar connectivity after SCI similarly reports strengthened coupling between cerebellar and brainstem motor centers in individuals with more severe motor impairment, and also reports that virtual-reality-based rehabilitation can recover structural volume, indicating neuroplastic potential [109]. Collectively, these observations indicate that disrupted cerebellar regulation of brainstem and spinal circuits amplifies motoneuron and reflex hyperexcitability after injury and represents an under-recognized supraspinal contributor—and potential therapeutic target—within the broader pathophysiology of spasticity.

### Treatment of Abnormal Tone and Spasticity

Given the multifactorial mechanisms underlying abnormal tone and spasticity after SCI, no single therapy is sufficient. Management is therefore multimodal, integrating rehabilitation, pharmacologic treatment, and, in refractory cases, surgical or neuromodulatory interventions. Established rehabilitative strategies—including stretching, range-of-motion exercises, task-specific practice, and weight-bearing—modulate segmental circuits by altering muscle spindle afferent input, reducing γ-motor activity, and inducing short-term depression of Ia-mediated reflexes [110]. Tendon vibration and sustained muscle loading further engage inhibitory pathways, acutely reducing tone. The central role of sensory afferents in sustaining spasticity is underscored by selective dorsal rhizotomy, an irreversible yet highly effective intervention that reduces severe spasticity by eliminating hyperactive sensory inflow [111].

Pharmacologic therapy remains the mainstay of systemic management and primarily targets excessive excitability in spinal circuits by modulating ion channels and neurotransmitters. Baclofen, the most widely used agent, is a GABA_B receptor agonist that suppresses neurotransmitter release from Ia afferents(presynaptic inhibition), reduces PIC amplification in motoneurons, and enhances both presynaptic and postsynaptic inhibition [112]. Although effective, oral baclofen is limited by sedation, fatigue, and weakness; intrathecal baclofen (ITB) allows target-specific delivery to spinal receptors at ~1/100th of the oral dose, reducing systemic adverse effects and providing potent suppression of spasms and clonus [113], although requiring consistent maintenance.

Spasticity after SCI is strongly influenced by upregulation of 5-HT_2_A/2C receptors that normally receive descending serotonergic input. Antagonists of these receptors—such as cyproheptadine, ritanserin, and ketanserin—can reduce motoneuron PICs and attenuate spasticity. Tizanidine, an α_2-adrenergic agonist, indirectly reduces 5-HT-mediated PIC amplification, reducing polysynaptic reflex transmission by inhibiting excitatory interneurons and decreasing facilitation from supraspinal centers [114]. Cyproheptadine is a potent 5-HT2 receptor antagonist that suppresses PICs but may have sedating and anticholinergic effects [115].

Diazepam, acting via GABA_A receptors, increases postsynaptic inhibitory conductance but is limited by tolerance, sedation, and cognitive effects. Dantrolene sodium acts peripherally by inhibiting ryanodine receptor–mediated calcium release in skeletal muscle, thereby reducing muscle contraction independently of spinal circuitry—useful when central agents are ineffective, but with risks of hepatotoxicity.

For focal or asymmetric patterns, botulinum toxin type A provides targeted chemo denervation with both peripheral effects (blocking acetylcholine release) and potential secondary central effects via altered afferent input [116]. Cannabinoids, particularly nabiximols, have shown modest benefit for spasticity in multiple sclerosis via CB1-mediated reduction in excitatory neurotransmission, though evidence in SCI remains limited [117]. Additional agents—gabapentinoids and clonidine—are used selectively based on symptom phenotype but have variable efficacy.

Despite this therapeutic armamentarium, current pharmacologic treatments largely suppress spasticity without reversing its underlying pathophysiology. Consequently, optimal management typically requires life-long combination therapy that integrates pharmacologic agents with rehabilitation, neuromodulation, and task-specific training to restore a more physiological balance of excitability across spinal and supraspinal networks.

## 8. Neuromodulatory Approaches

**Figure 2:**
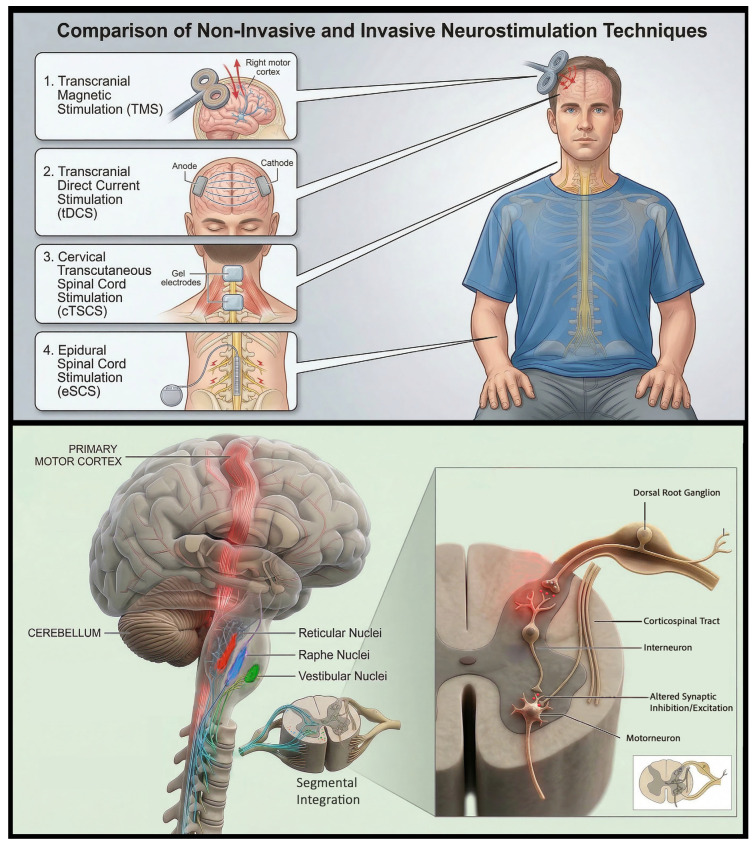
Upper panel, four methods of electrical stimulation to reduce spasticity and improve function are depicted. Lower panel, supraspinal and spinal segmental structures related to tone and spasticity include the motor cortex, cerebellum, and multiple brainstem nuclei, including the reticular, raphe, and vestibular systems. Lower right, an interneuron lying between sensory inputs and motor neuron output is depicted. Alterations in interneurons can lead to increased tone and spasticity through changes in presynaptic and post-synaptic inhibition. AI-assisted image generation was used solely for the creation of preliminary visual elements via the Artlist.io platform (Nano Banana Pro; Google Gemini-based image model). All scientific content, figure design decisions, annotations, and final image editing were performed by the authors using Adobe Photoshop 2026 27.5.0 and Adobe Illustrator 2026 (30.3).

Technically, almost all spasticity approaches are “neuromodulatory,” but there is significant interest in electrical current-based approaches to modulate circuits as a potential therapy, with a view to promoting corrective neuroplasticity (Figure 2).

## 9. Transcranial Direct Current Stimulation (tDCS)

Direct current stimulation (tDCS) is a non-invasive neuromodulation technique that applies low-intensity direct current (typically 1–2 mA) through scalp electrodes to modulate neuronal membrane potentials and alter cortical excitability [118]. Anodal stimulation generally depolarizes cortical neurons, increasing the likelihood of firing, whereas cathodal stimulation tends to hyperpolarize cortical tissue and reduce excitability [118]. When applied over the primary motor cortex (M1), tDCS can change corticospinal output [119], reorganize motor maps, and influence M1–brainstem interactions.

In the context of SCI, modulation of M1 excitability by tDCS may influence spasticity through several convergent mechanisms. First, augmenting corticospinal excitability can strengthen residual descending inhibitory control over spinal reflex circuits, a physiological effect supported by TMS studies showing that greater CST output is associated with lower reflex gain and reduced spasticity in both SCI and stroke populations [120,121]. Even modest increases in CST activity are mechanistically meaningful, as corticospinal projections exert inhibitory control over spinal interneuron networks and help restrain overactive reticulospinal pathways [90]. Second, anodal tDCS can enhance activation of M1 intracortical inhibitory circuits, including GABA_A_-mediated short-interval intracortical inhibition—changes that are known to downregulate descending facilitatory drive and modulate brainstem excitability [122,123]. Third, tDCS-induced increases in CST drive may improve voluntary recruitment of spinal inhibitory interneurons, thereby helping to counteract exaggerated PICs and reducing stretch reflex hyperexcitability.

Small clinical studies have reported modest reductions in spasticity following anodal M1 tDCS in individuals with SCI [124]. Although effects are transient, they appear stronger when paired with task-specific or repetitive motor training, likely reflecting synergistic plasticity in corticospinal and intracortical networks. Importantly, these results suggest that cortical modulation can shift the supraspinal balance toward greater inhibitory control of spinal circuits. Combined brain and spinal cord modulation has been reported using tDCS and peroneal nerve stimulation, with neurophysiological documentation of increased presynaptic and reciprocal inhibition [125]. While still preliminary, this line of work highlights M1-targeted stimulation as a potential adjunct to restore top-down regulation of reflex pathways and counteract the brainstem-driven hyperexcitability that underlies chronic spasticity.

## 10. Repetitive Transcranial Magnetic Stimulation (rTMS)

Repetitive transcranial magnetic stimulation (rTMS) is an established, non-invasive neuromodulation technique in which a rapidly changing magnetic field induces brief electric currents in cortical tissue, preferentially exciting axons of horizontally oriented pyramidal neuron axon segments in layers II/III and V as well as inhibitory interneurons [126,127]. Depending on coil orientation and intensity, rTMS can recruit CST neurons directly or via trans-synaptic activation of intracortical circuits. Clinically, rTMS is approved for several indications, including major depressive disorder and certain neuropathic pain syndromes, and its expanding use in neurorehabilitation has prompted investigation into its potential to modulate tone and spasticity after SCI [128].

rTMS uses trains of pulses delivered over defined cortical targets to induce lasting changes in excitability. High-frequency (>5 Hz) stimulation and facilitatory protocols such as intermittent theta-burst stimulation (iTBS) generally increase corticospinal excitability, whereas low-frequency (≤1 Hz) and continuous TBS tend to produce inhibitory effects, including long-term depression (LTD) [129,130]. In SCI, most studies have delivered excitatory rTMS over M1 of the more affected limb, aiming to augment residual corticospinal output and restore top–down control over brainstem and spinal circuits. High-frequency M1 rTMS has been reported to reduce spasticity and clonus in incomplete SCI, accompanied by increased motor evoked potentials and reduced H-reflex amplitudes, suggesting strengthened corticospinal drive and down-regulation of segmental reflex excitability. [124]. Similar protocols in multiple sclerosis have shown improvements in Modified Ashworth Scale scores and decreased H-reflex ratios, consistent with reduced α-motoneuron hyperexcitability [131].

Proposed mechanisms include modulation of intracortical inhibitory circuits, evidenced by increased short-interval intracortical inhibition (SICI) and changes in intracortical facilitation after facilitatory rTMS, which may normalize the imbalance between excitatory and inhibitory networks in M1 and reduce excessive descending drive onto brainstem premotor nuclei [128,130]. rTMS can also induce reorganization of motor maps, expanding the representation of the trained/spastic muscles and improving the specificity of corticospinal projections, thereby enhancing the voluntary recruitment of spinal inhibitory interneurons. Target selection appears important: stimulation over the ipsilesional M1 leg/arm hotspot or over premotor regions with strong connectivity to the affected muscles tends to produce more robust changes in corticospinal excitability than vertex or non-specific placements, and pairing rTMS with task-specific training likely leverages Hebbian plasticity to consolidate effects [132].

However, the current evidence base remains limited. Most studies involve small sample sizes, heterogeneous stimulation parameters, and short follow-up periods, with reported benefits typically lasting days to a few weeks and requiring repeated sessions for maintenance. Response appears to depend on the completeness, chronicity, and residual corticospinal integrity of the injury, and not all trials have reported significant clinical improvement [128]. Practical limitations include treatment burden, cost, restricted access to equipment, and a small but non-zero risk of inducing seizures with high-frequency protocols in susceptible individuals. Thus, rTMS should currently be viewed as a promising adjunct that can modulate supraspinal control of spasticity—particularly in people with preserved CST pathways—rather than a stand-alone or definitively proven anti-spasticity therapy.

### Comparison of rTMS and tDCS, Including Evidence for Combined Approaches

While both rTMS and tDCS aim to modulate cortical excitability and influence descending motor control, they differ substantially in biophysics, focality, and the degree to which they directly activate corticospinal pathways. tDCS delivers a weak, constant current that modulates neuronal membrane potentials without generating action potentials, producing broad, subthreshold shifts in the likelihood of neuronal firing. This global modulation can enhance intracortical inhibitory circuits and slightly increase residual CST output, but its impact on spinal circuits is modest and typically requires pairing with active training for durable changes. In contrast, rTMS induces direct neuronal depolarization via electromagnetic induction, preferentially exciting pyramidal neurons and trans-synaptic corticocortical and corticospinal circuits. High-frequency rTMS produces larger and more reliable increases in CST activity, alters motor map representations, and can rebalance supraspinal control by counteracting excess reticulospinal drive—mechanisms directly relevant to spasticity after SCI. Practically, tDCS is simpler and more accessible, whereas rTMS is more powerful, focal, and mechanistically aligned with the restoration of top-down inhibitory control.

Importantly, a growing body of work suggests that tDCS and rTMS can be combined to achieve additive or synergistic effects. Anodal tDCS delivered immediately before facilitatory rTMS has been shown to “prime” the cortex by shifting membrane potentials into a more excitable state, thereby enhancing the magnitude and duration of rTMS-induced plasticity [133]. In motor rehabilitation studies, combined anodal tDCS with iTBS or high-frequency rTMS has produced greater corticospinal gains and improved motor learning than either modality alone [134,135]. Although combined protocols have not yet been systematically tested for spasticity after SCI, the mechanistic rationale is compelling: tDCS can modulate the cortical “state,” while rTMS can induce synaptic plasticity that restores CST-mediated inhibition of spinal reflexes and reduces maladaptive reticulospinal facilitation. Given that chronic SCI spasticity reflects impaired CST influence and exaggerated brainstem drive, leveraging state-dependent metaplasticity through sequential tDCS + rTMS could represent a promising multimodal strategy for future investigation.

## 11. Epidural Spinal Cord Stimulation (eSCS)

Epidural spinal cord stimulation (eSCS) delivers continuous or patterned electrical pulses via small multi-contact electrodes implanted in the epidural space, typically over the dorsal roots and dorsal columns of the lumbosacral cord. eSCS is commonly used to treat chronic pain. Contemporary systems deliver charge-balanced biphasic or pseudo-monophasic pulses with programmable amplitude (typically in the mA or V range, depending on constant-current vs. constant-voltage mode), pulse width (~200–1000 μs), and frequency (30–80 Hz or 10 kHz). The combination of contact configuration, rostrocaudal position relative to dorsal root entry zones, pulse width, frequency, and amplitude strongly determines which neural elements are activated. Modeling and human recordings indicate preferential recruitment of large-diameter posterior-root afferents at clinically relevant intensities, rather than direct activation of motor axons in the cord [136,137,138].

Mechanistically, eSCS for SCI primarily engages posterior root–muscle (PRM) reflex pathways, producing trans-synaptic excitation of interneurons and motoneurons via these afferents, analogous to an H-reflex but with multi-root, multi-segment recruitment depending upon the selected parameters [138,139]. Neurophysiologic studies in chronic human SCI have shown that eSCS modifies spinal reflex behavior, including reductions in tonic EMG activity, attenuation of clonus, decreased H-reflex amplitudes, and changes in reflex recovery cycles—findings consistent with a net reduction in segmental hyperexcitability [136,138,139]. Work with both epidural and transcutaneous spinal stimulation has demonstrated enhanced presynaptic inhibition of Ia afferents (greater conditioning-induced H-reflex depression), partial restoration of reciprocal inhibition, and normalization of exaggerated PRM reflexes in individuals with SCI, supporting the idea that SCS can re-engage inhibitory interneuronal circuits that are underactive in spasticity [137,139,140].

Clinically, early studies in SCI reported that eSCS reduced spasticity in patients, with improvements on clinical spasticity scales, fewer flexor and extensor spasms, and easier passive movement during active stimulation [141]. Symptoms typically returned when stimulation was turned off [136,138]. Later studies, using careful parameter titration and precise placement over the upper lumbar segments, confirmed parameter-dependent control of tone, with optimal effects observed when electrodes were positioned over L1–L3 and frequencies in the ~50–100 Hz range, amplitudes of 2–7 V, and pulse widths of around 200 μs [138]. Locomotor-focused eSCS programs, such as those by Harkema and colleagues and subsequent cohorts, have also reported concurrent decreases in spasticity, as stimulation and task-specific training promoted more continuous, physiologic EMG patterns in lower-limb muscles during standing and stepping [137,142,143].

The magnitude and consistency of tone reduction depend strongly on stimulation parameters, electrode location, and residual descending inputs. Multi-contact mapping shows that small rostrocaudal or mediolateral shifts in active contacts can change which roots and interneuron circuits are engaged, producing different patterns of facilitation, co-contraction, or relaxation [137,142,143]. In incomplete injuries, where some corticospinal and reticulospinal fibers remain, eSCS appears to raise spinal network excitability to a state in which descending commands and afferent inputs are more effectively integrated, which may secondarily improve supraspinal control of tone. Reviews synthesizing this literature conclude that SCS can reduce spasms and hypertonia and improve other functions, such as pain and locomotion, although long-term, large-scale data on spasticity specifically remain limited [137].

Limitations include the surgical risks of electrode and pulse-generator implantation, hardware complications (infection, lead breakage, migration), and the challenge of achieving spasticity relief without uncomfortable paresthesias or interference with desired motor patterns. Postural changes and spinal alignment can alter the lead–cord distance and thus recruitment, contributing to variability in daily efficacy [137,138]. Moreover, most studies show that the anti-spasticity effects are state dependent—present during stimulation and decaying after cessation—indicating that, as currently implemented, eSCS modulates but does not reverse the underlying maladaptive plasticity. Nevertheless, converging clinical and neurophysiological evidence supports eSCS as a promising component of integrated strategies targeting both spasticity and motor function, particularly when combined with intensive, task-specific training and careful parameter optimization. Recently, high-frequency eSCS has been reported to potently reduce excessive tone and facilitate walking recovery when combined with lower-frequency stimulation to selectively activate muscles [144]. Eventually, it will be important to achieve closed-loop control of eSCS, e.g., by sensing tone and spasticity to regulate eSCS [145].

### Transcutaneous Spinal Cord Stimulation (TcSCS) and Spasticity

While eSCS, rTMS, and tDCS have all demonstrated the ability to reduce spasticity and modulate spinal and cortical excitability, each has limitations. eSCS requires invasive surgery, detailed programming, and hardware maintenance; rTMS and tDCS are non-invasive, require appropriate brain region targeting, and their effects are often transient, requiring repeated sessions with variable carryover. Most studies have examined lower limb tone and spasticity. These constraints underscore the need for simpler noninvasive, reproducible, and segment-specific neuromodulatory approaches that can simultaneously target local spinal circuits and supraspinal networks across different axial levels of the spinal cord.

Transcutaneous spinal cord stimulation (TcSCS) [146,147] is emerging as another neuromodulatory approach that can alter spasticity and heightened tone. Delivered through skin surface cathodal electrodes over the spine, TcSCS can produce a broad, multi-segmental current field that can engage large-diameter afferents, modulate segmental reflex excitability, and influence propriospinal and descending pathways [148,149,150]. When paired with intentional movement or task-specific rehabilitation, TcSCS may leverage Hebbian, spike-timing–dependent plasticity [151] to reinforce appropriate motor commands, strengthen inhibitory circuits, and suppress maladaptive reflex loops [152]. Early observations suggest that stimulation frequencies above ~50 Hz are particularly effective for modulating tone and spasticity during treatment sessions [153].

## 12. Segmental Effects of TcSCS

For cervical injuries, a notable feature of TcSCS is the placement of electrodes both above and at or below the injury site, allowing modulation of multiple segments and potential ascending stimulation to supraspinal centers. TcSCS can reduce motoneuronal hyperexcitability, lowering Hmax/Mmax ratios and improving Penn Spasm Frequency Scale and clonus scores [154,155]. It can improve postsynaptic reciprocal inhibition, normalize excessive heteronymous Ia facilitation, and may upregulate trophic factors such as BDNF [156]. Repeated TcSCS can reduce soleus H-reflex excitability and enhance spinal cord inhibition [154], suggesting partial restoration of inhibitory regulation. It is also possible to deliver TcSCS at multiple segmental sites to potentiate its effects [157].

## 13. Supraspinal Effects of TcSCS

TcSCS may activate ascending systems when electrodes are placed rostral to the injury, or when the injury is incomplete. Enhanced Event Related Desynchronization in the sensorimotor cortex during motor tasks has been observed [158], and increased activation in subcortical and cortical sensorimotor regions involved in relay and processing of movement-related somatosensory information [159]. At high intensities, TcSCS can produce cortical inhibition in healthy individuals [158].

Recent clinical work pairing TcSCS with brain–computer interface (BCI) motor imagery training further supports its supraspinal effects, showing that stimulation can sharpen sensorimotor cortical activation patterns, enhance cortical inhibition, and accelerate skill acquisition. Notably, the effects were site-specific: stimulation delivered above the lesion improved cortical ERD focality and BCI control, whereas stimulation below the lesion produced minimal supraspinal benefit [160]. These findings suggest that targeting stimulation sites with intact ascending pathways may be critical for maximizing supraspinal modulation.

Greater facilitation of cortical excitation or leg muscles has been demonstrated when TcSCS is combined with peripheral nerve stimulation pulses within the spike-dependent time window [161]. Representative studies of electrical neuromodulation are summarized in Table 1.

## 14. Plasticity Mechanisms

When recovery is observed either acutely during a neuromodulation session or cumulatively over repeated sessions, neuroplasticity is often invoked to explain it. While this is likely correct, cost-effective methods to directly interrogate underlying molecular mechanisms in humans remain limited [155]. Moreover, neuromodulation is unlikely to act exclusively on neural circuits; concurrent changes in muscle properties and other multisystem adaptations likely contribute to observed effects [164].

The neuroplastic effects of TcSCS remain relatively unexplored but may extend beyond transient modulation of excitability to induce more durable circuit-level changes with repetition. Reported mechanisms include alterations in pre- and postsynaptic inhibition [155], and animal studies suggest improvements in chloride homeostasis via KCC2. A key principle is that neuromodulation is most effective when paired with voluntary effort [165], and tonic stimulation combined with rehabilitation may also promote white matter plasticity [166].

## 15. Integration with Other Plasticity-Enhancing Therapies

Compared with pharmacological interventions, TcSCS and other forms of electrical neuromodulation avoid systemic side effects, preserve voluntary control, and prime circuits for physical therapy. Pairing TcSCS with plasticity-enhancing biologics, such as small molecules, antibodies, trophic proteins, or intermittent hypoxia, may amplify the potential for restorative neuroplasticity [167].

## 16. Discovery Methods for New Spasticity Therapies

A fundamental challenge in developing new therapies for elevated tone and spasticity is that these phenomena are circuit-emergent rather than attributable to any single molecular lesion. Spasticity arises from the cumulative loss of distributed inhibitory tone across multiple spinal laminae and interneuron classes —meaning that no individual target is likely to be sufficient, and that interventions must account for the reorganized state of the circuit at the time of treatment.

Emerging omics approaches—including bulk transcriptomics, single-cell RNA sequencing, proteomics, and spatial multi-omics—may provide a framework for resolving circuit complexity at cellular and laminar resolutions. Rather than treating spasticity as a unitary phenomenon, these methods can help identify the specific cell types and molecular pathways that drive hyperexcitability, loss of inhibition, maladaptive synaptic plasticity, neuroinflammation, and muscle fibrosis after spinal cord disorders.

Omics studies in SCI have begun to define distinct injury microenvironments across time and space and to reveal potential therapeutic pathways [168]. Although their application to spasticity remains early, transcriptomic analyses have already provided key insights. In particular, bulk RNA sequencing has identified downregulation of the potassium–chloride cotransporter KCC2 (SLC12A5) in motoneurons as one of the most robust molecular correlates of spasticity, highlighting disrupted chloride homeostasis as a tractable therapeutic target.

Single-cell and single-nucleus RNA sequencing provide high-resolution profiling of inhibitory interneuron populations, enabling cell-type–specific analysis of spinal inhibitory circuits. Recent atlases of the adult human spinal cord integrating snRNA-seq with spatial transcriptomics have resolved the laminar organization and molecular identities of GABAergic and glycinergic neurons in the ventral horn, creating a framework for mapping injury-induced transcriptional changes [169]. Spatial transcriptomics is particularly valuable for spasticity research because it allows direct comparison of lamina-specific alterations, such as differences between lamina VII interneurons (including Renshaw cells and Ia inhibitory interneurons) and lamina IX motor pools. However, most studies to date rely on rodent models at fixed post-injury timepoints, and human tissue from individuals with chronic spasticity remains limited. Cell-free RNA in CSF and exosomal RNA from SCI patients stratified by spasticity severity may help bridge this translational gap.

Epigenetic mechanisms may also contribute to the persistence of elevated tone and spasticity after SCI by inducing durable changes in gene regulation, including DNA methylation, histone modification, and non-coding RNA signaling [170]. These processes may reinforce spasticity by sustaining neuroinflammation, disrupting inhibitory neurotransmission, and altering chloride homeostasis, including KCC2 expression [171]. Rodent studies demonstrate methylation-mediated silencing of the SLC12A5 promoter and increased repressive histone marks (e.g., H3K27me3) at inhibitory synapse gene loci, which may underlie the progressive loss of inhibitory interneuron function after injury. Consistent with this model, HDAC inhibition restores GAD65 and KCC2 expression in spinal hyperexcitability models [172], suggesting that epigenetic regulation helps maintain maladaptive, hyperexcitable circuit states over time. Electrically induced exercise, however, can produce favorable epigenetic changes in muscles [173].

Translating these molecular discoveries into effective therapies presents several interrelated challenges. Because spasticity arises from distributed, circuit-level dysfunction rather than a single focal molecular lesion, transcriptomic and epigenetic targets identified in animal models often exhibit diffuse expression patterns, complicating therapeutic specificity. Central nervous system delivery represents an additional barrier, as many biologic approaches—including antisense oligonucleotides and gene therapy vectors—are likely to require intrathecal administration to achieve adequate concentrations at relevant spinal segments. Moreover, the timing of intervention is critical: strategies aimed at preventing epigenetic silencing of inhibitory pathways in the early post-injury period are mechanistically distinct from those intended to restore inhibition within a chronically reorganized circuit. These temporal differences have important implications for trial design, patient stratification, and outcome selection.

## 17. Summary

Abnormal muscle tone and spasticity after SCI emerge from distributed changes across spinal and supraspinal motor systems rather than from a single pathological mechanism. Injury disrupts descending inhibitory control while simultaneously triggering adaptive and maladaptive plasticity within spinal circuits, including motoneurons, interneurons, and sensory afferents. These changes are reinforced by alterations in muscle properties, sensory feedback, and brain–brainstem regulatory systems, creating self-sustaining reflex loops that degrade voluntary control and functional mobility.

Current pharmacologic therapies can reduce symptoms but generally do not correct the underlying circuit dysregulation that drives persistent hyperexcitability. Neuromodulatory approaches—including epidural stimulation, rTMS, and transcranial direct current stimulation—demonstrate that targeted modulation of spinal and supraspinal networks can influence tone regulation, but these strategies remain limited by invasiveness, variability of response, or transient effects.

Transcutaneous spinal cord stimulation (TcSCS) is a promising noninvasive neuromodulatory strategy that can engage segmental reflex pathways, propriospinal circuits, and supraspinal control networks simultaneously. When combined with task-specific rehabilitation, TcSCS may promote adaptive neuroplasticity, restoring a more physiological balance between excitation and inhibition. Future progress will depend on defining optimal stimulation paradigms, identifying biomarkers that reflect circuit state, and integrating neuromodulation with pharmacologic, biologic, and omics-guided therapies to develop mechanism-based treatments for spasticity after SCI.

## 18. Conclusions

- Spasticity after spinal cord injury reflects distributed dysfunction across spinal and supraspinal motor systems rather than a single reflex abnormality.
- Segmental mechanisms include motoneuron persistent inward currents, interneuron phenotype switching, sensory afferent hyperexcitability, synaptic reorganization, and structural muscle remodeling.
- Supraspinal contributors include cortical motor map reorganization, reduced intracortical inhibition, corticospinal–reticulospinal imbalance, and loss of monoaminergic modulation of spinal circuits.
- Neuromodulatory approaches can influence both spinal and supraspinal networks, restoring inhibitory control and rebalancing excitatory–inhibitory circuit dynamics.
- Transcutaneous spinal cord stimulation (TcSCS) offers a non-invasive strategy to modulate spinal reflex circuits and descending control pathways and may promote adaptive neuroplasticity when combined with task-specific rehabilitation.
- Emerging transcriptomic and epigenetic approaches may enable mechanism-based therapies and biomarker-guided strategies for treating spasticity after SCI.

## Figures and Tables

**Table 1:** Standardized summary of representative human SCI neuromodulation studies. Includes studies directly evaluating spasticity/tone as well as pivotal clinical SCI trials relevant to the maturation of each modality.

Modality	Study (Year)	Study Type	SCI Population	N	Stimulation Parameters	Outcome Measures (Spasticity/Tone)	Follow-Up/Durability	Key Findings/Interpretation
tDCS	Yamaguchi et al. (2016) [125]	Single-masked crossover	Chronic incomplete SCI (AIS C/D)	11	Anodal M1, 1 mA, 20 min + peripheral stimulation	Reciprocal inhibition, kinematics	Immediate	Indirect evidence for spasticity modulation
tDCS	Chantanachai et al. (2025) [162]	Randomized sham-controlled	Chronic SCI, mixed severity	30	Repeated sessions over 4 weeks	Clinical spasticity scales	1 month	No significant benefit vs. sham
rTMS	Kumru et al. (2010) [124]	Controlled study	Incomplete SCI with spasticity	22	20 Hz M1 stimulation, 5 days	MAS, VAS, SCI-SET	1 week	Reduced spasticity in active group
rTMS	Benito et al. (2012) [163]	Prospective	Incomplete SCI	17	High-frequency M1 rTMS	MAS, reflex measures	Short-term	Improved tone; requires repetition
eSCS	Pinter et al. (2000) [138]	Interventional	Chronic SCI with severe spasticity	8	L1–L3, 50–100 Hz, 2–7 V	Clinical + EMG	During stimulation	Strong state-dependent reduction
eSCS	Harkema et al. (2011) [142]	Case study	Motor-complete SCI	1	Lumbosacral stimulation	EMG patterns	During sessions	Improved motor patterns
TcSCS	Hofstoetter et al. (2014) [139]	Pilot	Motor-incomplete SCI	3	T11–T12, ~50 Hz, 30 min	Reflex + EMG	Immediate	Reduced reflex overactivity
TcSCS	Hofstoetter et al. (2020) [140]	Cohort	Mixed SCI	12	Lumbar ~50 Hz, 30 min	MAS, clonus	2 h	Short-term and cumulative effects
TcSCS	Moritz et al. (2024)—Up-LIFT [147]	Multicenter trial	Chronic cervical SCI	60	30 Hz + 10 kHz carrier, ~25 sessions	Functional outcomes	2 months	Strong clinical efficacy, indirect tone relevance

Abbreviations: tDCS, transcranial direct current stimulation; rTMS, repetitive transcranial magnetic stimulation; eSCS, epidural spinal cord stimulation; TcSCS, transcutaneous spinal cord stimulation; MAS, Modified Ashworth Scale.

## Data Availability

No original data were used for this review.

## Abbreviations

Abbreviation	Definition
AMPA	α-amino-3-hydroxy-5-methyl-4-isoxazolepropionic acid receptor
AMPAR	AMPA receptor
BCI	Brain–Computer Interface
BDNF	Brain-Derived Neurotrophic Factor
BMD	Bone Mineral Density
CGRP	Calcitonin Gene-Related Peptide
CNS	Central Nervous System
COL1A1	Collagen Type I Alpha 1 Chain
CST	Corticospinal Tract
DRG	Dorsal Root Ganglion
ECM	Extracellular Matrix
ERD	Event-Related Desynchronization
FES	Functional Electrical Stimulation
GABA	Gamma-Aminobutyric Acid
GAD67	Glutamate Decarboxylase 67
HO	Heterotopic Ossification
KCC2	Potassium-Chloride Cotransporter 2
LTD	Long-Term Depression
LTP	Long-Term Potentiation
MRI	Magnetic Resonance Imaging
NMDA	N-Methyl-D-Aspartate receptor
PI3K	Phosphoinositide 3-Kinase
PSFS	Penn Spasm Frequency Scale
RANKL	Receptor Activator of Nuclear Factor κB Ligand
ROM	Range of Motion
RST	Reticulospinal Tract
SCI	Spinal Cord Injury
SICI	Short-Interval Intracortical Inhibition
SMA	Supplementary Motor Area
tDCS	Transcranial Direct Current Stimulation
TcSCS	Transcutaneous Spinal Cord Stimulation
TGFβ1	Transforming Growth Factor Beta 1
TMS	Transcranial Magnetic Stimulation
rTMS	Repetitive Transcranial Magnetic Stimulation
TNF-α	Tumor Necrosis Factor Alpha
PIC	Persistent Inward Current
mTOR	Mechanistic Target of Rapamycin
5-HT	5-Hydroxytryptamine (Serotonin)
